# Differential effects of rapid or slow body weight loss on muscle weight and protein degradation pathways in rat skeletal muscle

**DOI:** 10.1186/1550-2783-12-S1-P58

**Published:** 2015-09-21

**Authors:** Yudai Nonaka, Shin Terada

**Affiliations:** 1 Department of Life Sciences, Graduate School of Arts and Sciences, The University of Tokyo, 3-8-1 Komaba, Meguro-ku, Tokyo 153-8902, Japan

## Background

Many athletes restrict energy intake to achieve a certain body mass category, aesthetic benefits or to attain a better force-to-mass ratio to improve performance. This rapid weight loss, also known as "weight cutting", usually involves several-day fasting until a target weight is achieved. However, fasting is a well-known stimulus to activate two major protein degradation pathways, the autophagy-lysosome and ubiquitin-proteasome systems, resulting in skeletal muscle atrophy. An alternative dietary weight loss approach commonly practiced by athletes is daily caloric restriction, which consists of decreasing energy intake by 10-30% every day, resulting in slower body weight loss compared with fasting. The caloric restriction-induced slower body weight loss has also been shown to induce skeletal muscle atrophy. Since no study has directly compared the effects of rapid vs. slow body weight loss on muscle weight and the major protein degradation pathways during an equivalent body weight loss, it still remains unclear which rapid or slow weight loss approach is effective in maintaining skeletal muscle mass. The purpose of this study was thus to assess the effects of rapid or slow body weight loss on muscle weight and the protein degradation pathways in skeletal muscle.

## Methods

Twenty-week-old male Fischer rats were divided into the following 3 groups; fed ad libitum for 2 weeks (Control); subjected to 30% calorie restriction to decrease body weight slowly (Slow); or fasted for the last 3 days to rapidly decrease body weight comparable to that of the Slow group (Rapid). Fast-twitch plantaris and slow-twitch soleus muscles were dissected out and the expression ratio of autophagosomal membrane protein LC3-II to LC3-I and poly-ubiqutinated protein concentration were determined as biomarkers of the autophagy-lysosome and ubiquitin-proteasome activities, respectively.

## Results

Body weight and total intra-abdominal fat mass in the Slow and Rapid groups decreased to the same extent, although food intake was significantly higher in the Rapid than Slow group. Although muscle weight and muscle protein content of soleus muscle did not differ among the 3 groups, those of plantaris muscle were significantly lower in the Rapid but not in Slow group, compared with the Control group. Substantial increases in LC3-II protein expression, the ratio of LC3-II/-I and poly-ubiqutinated protein concentration were observed in plantaris muscle of the Rapid group. Moreover, the LC3-II/-I ratio or poly-ubiqutinated protein concentration was highly negatively correlated with the muscle weight or muscle protein content of plantaris muscle.

## Conclusion

These results suggest that rapid body weight loss by short-term fasting may induce muscle atrophy in fast-twitch muscle, at least in part through enhanced protein degradation by the autophagy-lysosome and ubiquitin-proteasome systems.

